# Supplementary material for: Carbohydrates complement high‐protein diets to maximize the growth of an actively hunting predator
Source: Ecol Evol. 2022 Jul 29;12(8):e9150. doi: 10.1002/ece3.9150 (PMC9336175; doi:10.1002/ece3.9150)
Supplement: Supplementary file 1 — Figure S1 [file ECE3-12-e9150-s001.pdf]

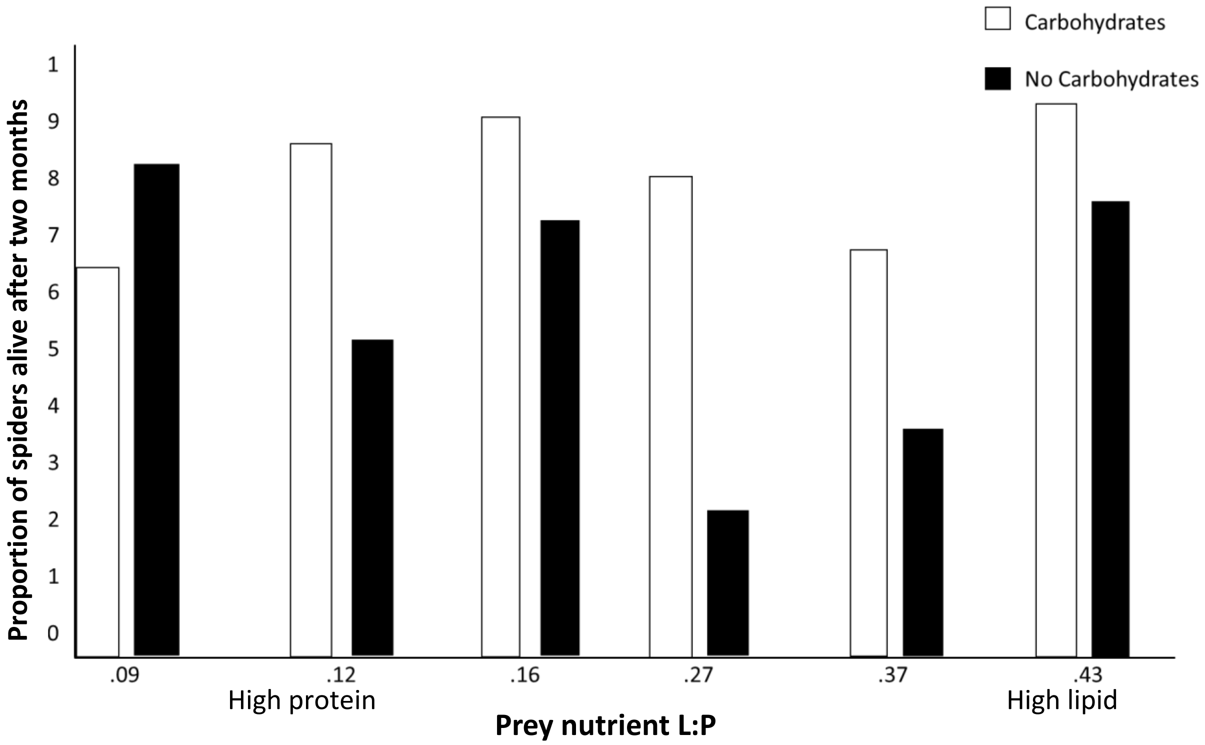

Figure S1. Parametric Survival of spiders across prey nutrients with and without supplemental carbohydrates.
